# Supplementary material for: High Cysteinyl Leukotriene Receptor 1 Expression Correlates with Poor Survival of Uveal Melanoma Patients and Cognate Antagonist Drugs Modulate the Growth, Cancer Secretome, and Metabolism of Uveal Melanoma Cells
Source: Cancers (Basel). 2020 Oct 13;12(10):2950. doi: 10.3390/cancers12102950 (PMC7600582; doi:10.3390/cancers12102950)
Supplement: Supplementary file 1 [file cancers-12-02950-s001.pdf]

cancers

Supplementary Materials: High Cysteinyl Leukotriene Receptor 1 Expression Correlates with Poor Survival of Uveal Melanoma Patients and Cognate Antagonist Drugs Modulate the Growth, Cancer Secretome and Metabolism of Uveal Melanoma Cells

Kayleigh Slater, Aisling B. Heeran, Sandra Garcia-Mulero, Helen Kalirai, Rebeca Sanz-Pamplona, Arman Rahman, Nebras Al-attar, Mays Helmi, Fiona O'Connell, Rosa Bosch, Anna Portela, Alberto Villanueva, William M. Gallagher, Lasse D. Jensen, Josep M. Piulats, Sarah E. Coupland, Jacintha O'Sullivan and Breandán N. Kennedy

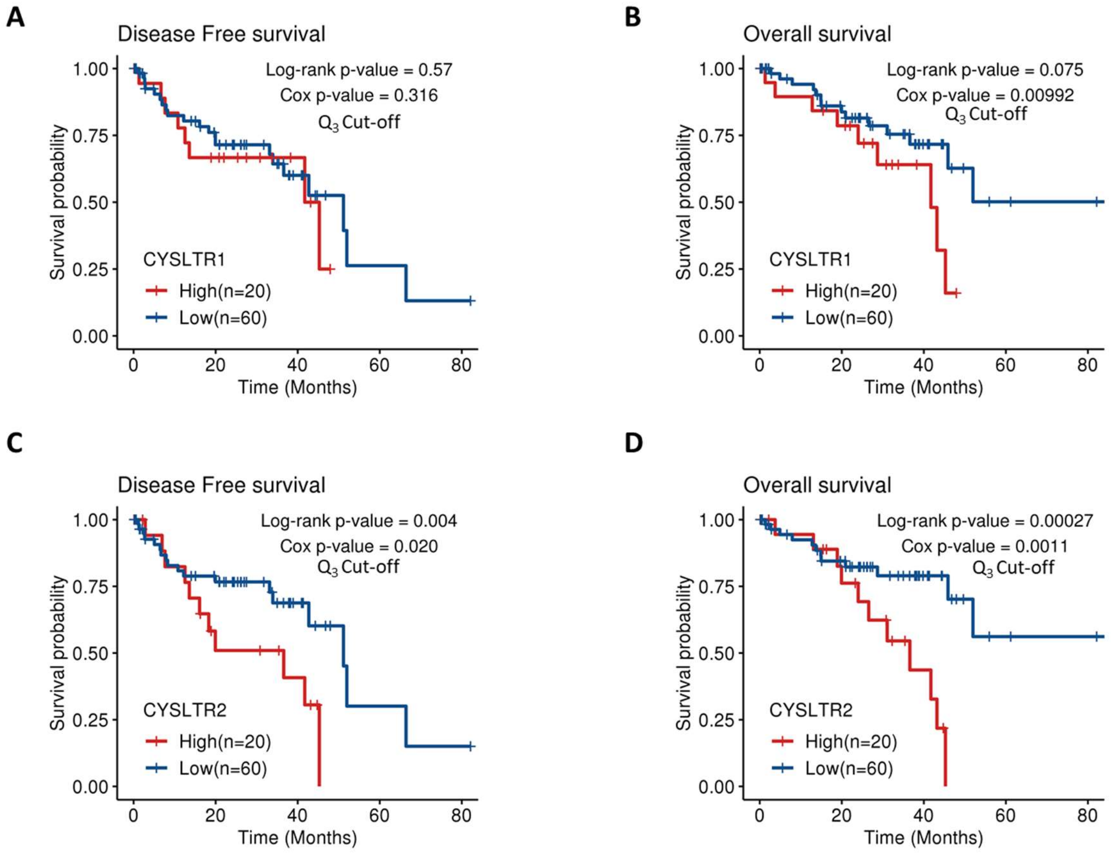

Figure S1. Analysis of *CYSLTR1*/*CYSLTR2* expression and UM patient survival from The Cancer Genome Atlas (TCGA) adjusted by ESTIMATE stromal score. Survival curves in Figure 1 were adjusted by stromal infiltration. When the models are adjusted by stromal scores, there is no statistically significant relationship between high (red) *CYSLTR1* expression and disease-free survival (**A**) or overall survival (**B**) (p = 0.57 and p = 0.075, respectively). High expression of *CYSLTR2* (red) maintains a statistically significant relationship with disease-free survival (**C**) and overall survival (**D**) when the models are adjusted by stromal cell infiltration (p = 0.004 and p = 0.00027, respectively).

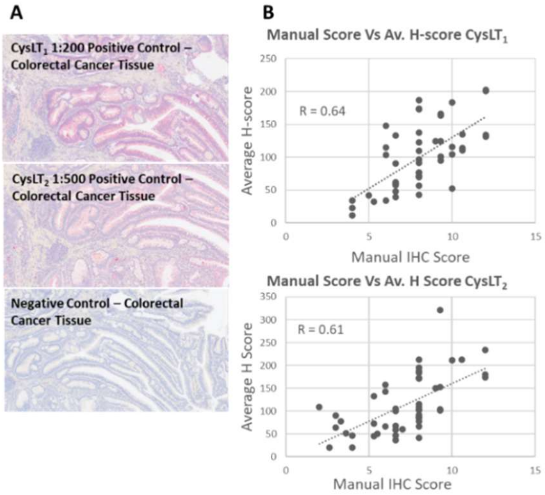

C

	Age at primary management	Largest ultrasound diameter	Ultrasound height	Mitotic count	Survival (Years) or to study end
Mean	62.2	17.03	9.2	6.7	8.49
Median	62.3	17.35	8	4	8
Range	50	13	13	31	18.6
Maximum	39	10.6	5	0	0.4
Minimum	89	23.6	18	31	19

D

Clinical Feature	Absent	Present	Unknown	Total number of patients	Association with high $CysLT_1$ expression (p – value)	Association with high $CysLT_2$ expression (p – value)
Extraocular extension	49	3	0	52	0.963	0.11
Monosomy 3	28	23	1	52	0.187	0.889
PAS positive loops	15	37	0	52	0.453	0.887
Ciliary body involvement	37	15	0	52	**0.041***	0.285
Epithelioid cell involvement	22	30	0	52	0.424	0.392

* $p < 0.05$

Figure S2. Patient TMA control tissue. Core-core correlations of manual versus digital pathology analysis. Clinical characteristics of patients included in the TMA. (**A**) Colorectal cancer tissue positive control for $CysLT_1$. Colorectal cancer tissue positive control for $CysLT_2$. Negative control (omission of antibody). (**B**) Correlation between manual score and the average H-score assigned by digital pathology analysis for $CysLT_1$ (R = 0.64) and $CysLT_2$ (R = 0.61). (**C**) Clinical characteristics of the patients included in the TMA analysed. (**D**) The relationship between prognostic clinical characteristics in UM and the expression of high $CysLT_1$ or $CysLT_2$. Ciliary body involvement has a statistically significant relationship with high $CysLT_1$ expression (p = 0.041) as assessed by Pearson's Chi-Square test.

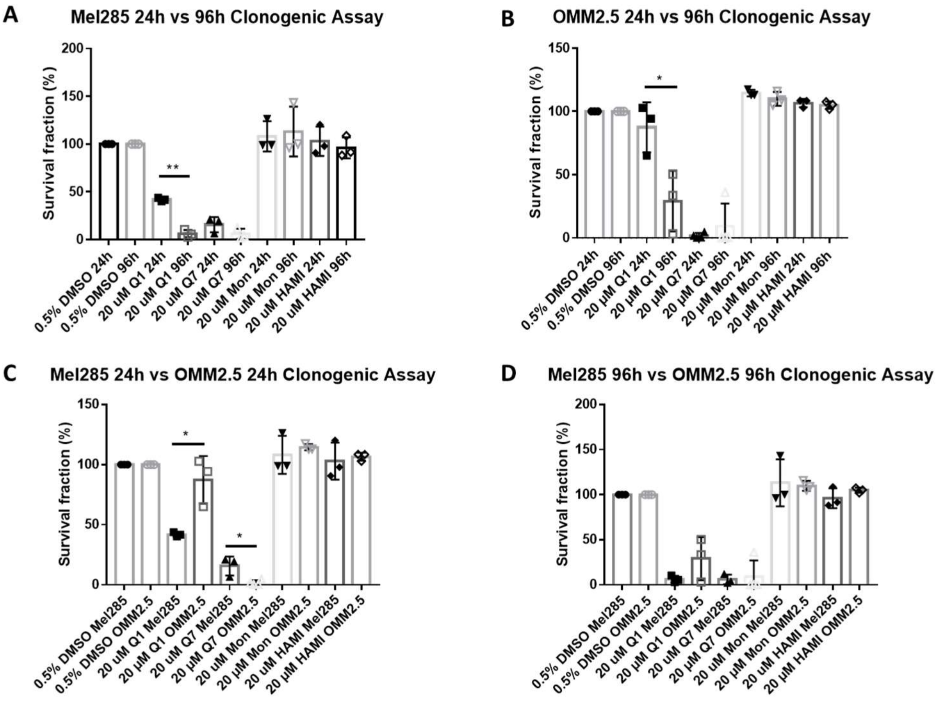

Figure S3. Cell line comparison data for the clonogenic assay. (**A**) Differences in the percentage survival fraction of Mel285 clones when treated for 24 versus 96 hours. Treatment with 20 µM quininib (Q1) is significantly more effective at 96 hours. (**B**) Differences in the percentage survival

fraction of OMM2.5 clones when treated for 24 versus 96 h. Treatment with 20 µM quininib (Q1) is significantly more effective at 96 h. (**C**) Differences in the percentage survival fraction of Mel285 versus OMM2.5 clones when treated for 24 h. 20 µM quininib (Q1) is significantly more effective in Mel285 cells following 24-hour treatment. While 20 µM 1,4–dihydroxy quininib (Q7) is significantly more effective in OMM2.5 cells following 24-hour treatment. (**D**) Differences in the percentage survival fraction of Mel285 versus OMM2.5 clones when treated for 96 hours. There were no statistically significant differences between cell lines following 96-hour treatment. Statistical analysis was performed using a paired t-test to compare within the same cell lines. An unpaired t-test was used to compare between different cell lines. Error bars are mean + S.E. * $p < 0.05$; ** $p < 0.01$.

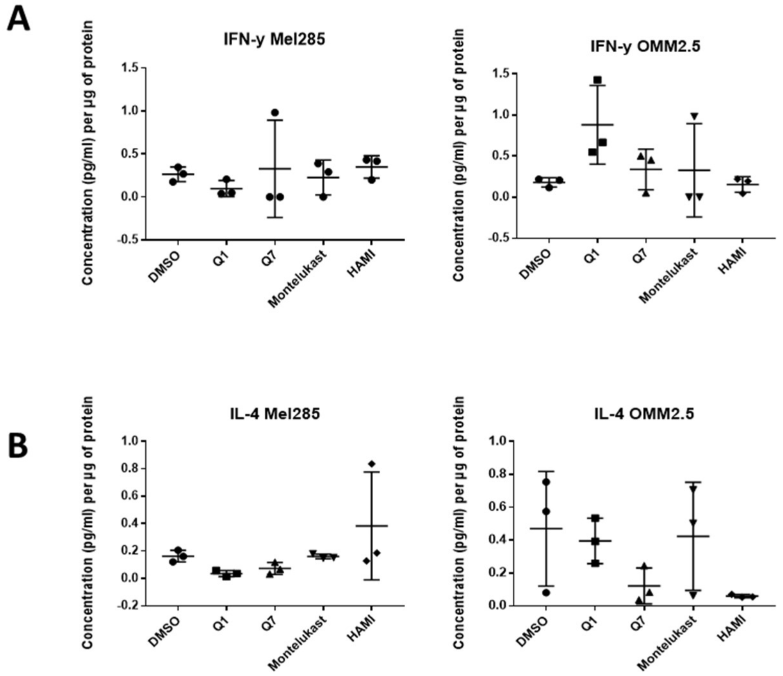

Figure S4. Factors unchanged by ELISA following 24 h treatment in Mel285 and OMM2.5 cells. Treatment with all CysLT targeting drugs tested (quininib (Q1), 1,4 –dihydroxy quininib (Q7), montelukast and HAMI 3379 (HAMI)) had no effect on the secretion of IFN- γ (**A**) or IL-4 (**B**) in Mel285 or OMM2.5 cells.

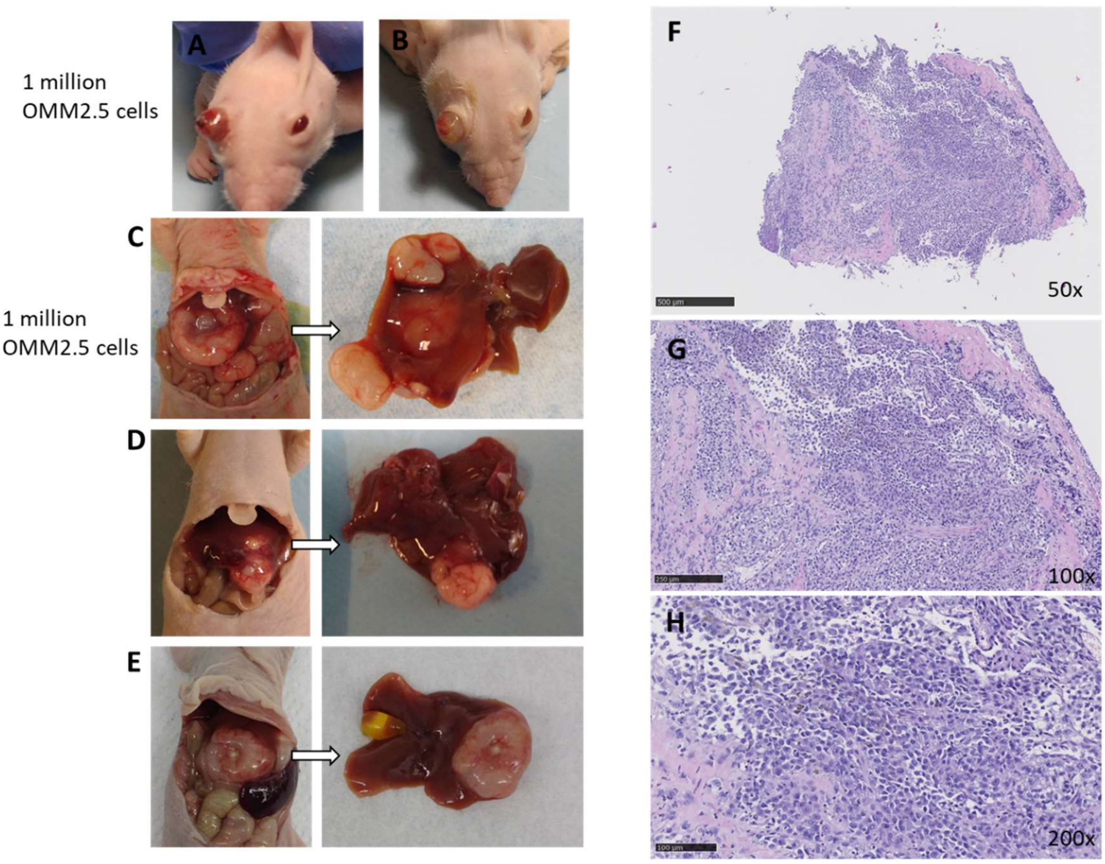

Figure S5. Generation of OMM2.5 cell line-derived orthotopic xenograft models of UM. Macroscopic appearance of an ocular tumour (**A**) 104 days after intraocular injection of 1 million OMM2.5 cells, or (**B**) 102 days after intraocular injection of disaggregated cells from ocular tumour (**A**). Macroscopic appearance and liver gross pathology of two representative mice injected with 1 million OMM2.5 cells in the liver, 98 days post-injection (**C**), 112 days post-injection(**D**), and of one representative mouse directly implanted with a OMM2.5 cell line-derived tumour fragment in the liver (**E**), 61 days post-implantation (**E**). H&E staining in representative sections of an ocular tumour from an OMM2.5 cell line- derived xenograft model. Original magnification ×50 (**F**), ×100 (**G**), ×200 (**H**).

Table S1. The association between *CYSLTR1* and *CYSLTR2* gene expression from TCGA and prognosis were assessed by Cox proportional hazard regression models (**A**,**B**,**C**). To resolve a violation of the proportional hazard assumption, the association between *CYSLTR2* gene expression and overall survival (**D**) was stratified by time from 0–20 months and beyond 20 months and analysed by the likelihood ratio (LHR) test. The hazard ratios and corresponding confidence intervals for *CYSLTR1*, *CYSLTR2*, sex, and age are reported.

**(A) Disease-Free Survival *CYSLTR1***			
Variable	HR	95% CI	*p*-value
Sex (male)	0.82	[0.44;1.20]	0.61
Age	1.03	[1.01;1.05]	0.09
CYSLTR1 (continuous)	1.05	[1.03;1.07]	0.01
Likelihood ratio test	-	-	0.06
**(B) Overall Survival *CYSLTR1***			
Variable	HR	95% CI	*p*-value
Sex (male)	1.47	[1.02;1.91]	0.39
Age	1.05	[1.02;1.07]	0.02
CYSLTR1 (continuous)	1.06	[1.04;1.08]	0.005
Likelihood ratio test	-	-	0.004
**(C) Disease-Free Survival *CYSLTR2***			

Variable	HR	95% CI	*p*-value
Sex (male)	0.73	[0.35;1.11]	0.41
Age	1.03	[1.01;1.04]	0.12
CYSLTR2 (continuous)	1.35	[1.25;1.45]	0.004
Likelihood ratio test	-	-	0.01
**(D) Overall Survial *CYSLTR2***			
Variable	HR	95% CI	*p*-value
Sex (male)	1.33	[0.89;1.77]	0.52
Age	1.05	[1.03;1.07]	0.02
CYSLTR2.adj:strata(tstart) tstart=0	1	[0.99;1.01]	0.47
CYSLTR2.adj:strata(tstart) tstart=20	1.01	[1;1.02]	0.0002
Likelihood ratio test	-	-	0.00003

© 2020 by the authors. Licensee MDPI, Basel, Switzerland. This article is an open access article distributed under the terms and conditions of the Creative Commons Attribution (CC BY) license (http://creativecommons.org/licenses/by/4.0/).
